# National Identification Counteracts the Sedative Effect of Positive Intergroup Contact on Ethnic Activism

**DOI:** 10.3389/fpsyg.2017.00477

**Published:** 2017-04-10

**Authors:** Adrienne Pereira, Eva G. T. Green, Emilio Paolo Visintin

**Affiliations:** Laboratory of Social Psychology, Institute of Psychology, Faculty of Social and Political Sciences, University of Lausanne,Lausanne, Switzerland

**Keywords:** intergroup contact, minority activism, ethnic identification, national identification, Roma

## Abstract

Positive intergroup contact with socially and economically advantaged national majorities has been shown to reduce ethnic identification among minorities, thereby undermining ethnic minority activism. This finding implies that ethnic identity is the relevant social identity driving ethnic minorities’ struggle for equality. We argue that the study of the “sedating” effect of positive intergroup contact for minorities should be more nuanced. The existence of multiple and sometimes interplaying social identities can foster a reinterpretation of the meaning of “ethnic” activism. This study therefore examines how the interplay of ethnic and national identities shapes the sedating effect of contact on minority activism. We expect national identification to buffer the sedated activism resulting from reduced ethnic identification. That is, the mediation from intergroup contact to reduced ethnic activism through weakened ethnic identification is expected to be moderated by national identification. With survey data from Bulgaria, we investigated support for ethnic activism among Bulgarian Roma (*N* = 320) as a function of their contact with the national majority as well as their degree of ethnic and national identification. The predicted moderated mediation was revealed: a negative indirect relationship between contact and activism through decreased ethnic identification occurred among Roma with low national identification, whereas no sedating effect occurred among Roma identifying strongly as members of the Bulgarian nation. We discuss the meaning of national identification for the Roma minority, who experience harsh discrimination in countries where they have been historically settled, as well as convergence of these findings with work on dual identification. We highlight the role of interacting social identities in mobilizing resources for activism and the importance of adopting a critical view on ethnic discourse when studying activism in both traditional and immigrant minorities.

## Introduction

Minority activism aims at modifying norms or practices established by a majority group (Moscovici, 1976). In the case of *ethnic* minority activism, unequal treatment and discrimination based on the ethnic categorization of individuals is challenged. Ethnic categories and the related social identities develop when a group of individuals share a common ancestry, physical traits or values differentiating them from others (Smith, 1991). Most countries are ethnically diverse in this sense, and ethnic identities usually differentiate subordinate minorities from dominant majorities within superordinate nation-states (see Staerklé et al., 2010). The ethnic identities of national minorities (or majorities) are indeed in part relational, stemming from interdependent comparisons, and frequently, unequal treatment.

Notwithstanding the criticism addressed to integration policies that focus on prejudice reduction rather than addressing structural inequalities, recent research has shown that members of ethnic minorities who experience positive contact with members of the advantaged, or dominant, majority display attenuated ethnic activism (e.g., Dixon et al., 2007), partly because of reduced ethnic identification (e.g., Wright and Lubensky, 2009). Building on the literature revealing this “irony of harmony” resulting from positive intergroup contact (Saguy et al., 2009; see however Kauff et al., in press), our goal is to examine whether the sedating effect of positive contact on ethnic activism via reduced ethnic identification is buffered by national identification of minority members. As national identities are central in the contemporary world (Reicher and Hopkins, 2001), we argue that the synergy between ethnic and national identifications in ethnic minorities is at play in intergroup encounters. The contribution of this research is twofold. First, we provide novel insights that speak to the recent integration of two research traditions—work on social identity predictors of activism and work on demobilizing effects of intergroup contact (see also, e.g., Çakal et al., 2011). Second, we examine the perspective of the Roma minority, historically one of the most severely rejected ethnic minorities in Europe (e.g., Heath and Richards, 2016), yet hardly studied in social psychology. We conducted a cross-sectional survey in Bulgaria, a multicultural society composed of ethnic minorities among which Roma are the second largest.

### Positive Intergroup Contact and Minority Activism

There is ample evidence that positive intergroup contact improves intergroup attitudes (Pettigrew and Tropp, 2006). In particular, individuals from advantaged groups who have positive contact experiences with members of disadvantaged social groups show less prejudice and negative emotions as well as greater support for egalitarian policies (e.g., Pettigrew and Tropp, 2011). However, the consequences of positive intergroup contact are different for members of disadvantaged groups. Recent research has revealed that positive contact with members of socially and economically advantaged groups is associated with attenuated support for egalitarian policies. For example, studies conducted in post-apartheid South Africa have shown that quantity of positive contact with Whites was related to Blacks’ decreased support to compensatory and preferential policies aiming to ensure racial equality (Dixon et al., 2007; Çakal et al., 2011) and reduced behavioral intentions in favor of the Black minority (such as signing a petition or participating in anti-discrimination projects; Çakal et al., 2011). Similarly, Tropp et al. (2012) revealed that ongoing friendships with Whites were associated with a progressive decline of support for ethnic activism among African- and Latino-American college students. In short, unless the struggle for equality is carried out by the advantaged majority, positive contact with majority members can demobilize minorities, and unintendedly result in the status quo of power relations.

Scholars have explored the psychological processes underlying this demobilization of minority members. For example, Saguy et al. (2009) found a relationship between experiencing positive contact with Israeli Jews and reduced support for social change among Israeli Arabs (i.e., improvement of their position in Israel). The relationship was mediated by improved attitudes toward Jews, by increased perception that Jews treat Arabs fairly, as well as by decreased awareness of structural inequalities (see also Çakal et al., 2011; Tropp et al., 2012 for similar findings showing decreased perceptions of discrimination). Furthermore, using self-reports of past interracial contact, Wright and Lubensky (2009) demonstrated that interactions with Whites before entering university reduced endorsement of ethnic-based collective action both in African- and Latino-American students. Crucial to the present study, the sedating effect of positive intergroup contact was mediated by reduced ethnic identification. Tausch et al. (2015) found a similar disidentification process among Latino-American students resulting from *present* interethnic friendships with Whites. In the current study, we thus also expect that positive intergroup contact with the Bulgarian national majority is related to reduced ethnic activism among the Bulgarian Roma minority through diminished ethnic identification.

The rationale for the sedative effect of contact put forward by Wright and Lubensky (2009) derives from the idea that collective action and prejudice reduction are two incompatible routes in disadvantaged groups’ struggle for social equality. Indeed, with ethnic identification driving ethnic activism, social protest implies recognizing social disadvantages and motivation to improve the status of the ingroup. Positive intergroup contact, in turn, results in lowered attention to inequalities (Saguy et al., 2009) and weakened salience of group categories (see Brown and Hewstone, 2005 for a discussion on category salience during intergroup contact). Experiencing positive contact with more advantaged individuals makes intergroup boundaries seem more permeable. Hence, the advantaged social identity becomes relevant and the disadvantaged (ethnic) identity less relevant for disadvantaged individuals. Consequently, members of disadvantaged groups reinterpret their social identity as mirroring a common ingroup shared with the advantaged group or as a dual identity with elements of the disadvantaged and advantaged identity (Gaertner and Dovidio, 2000). According to Wright and Lubensky (2009), in such identity configurations the subordinate ethnic identity is no longer strong enough to drive social protest, since it is dominated by or at least predisposed toward the advantaged group. Building on this research, in the current study, we suggest that the motivation to enhance the subordinate group’s position can remain in new identity reconfigurations following intergroup contact experiences and thereby allow for activism.

Dynamic reconfigurations of identity are not a new idea. Social identity theory (Tajfel and Turner, 1979) and the literature on social stigma (e.g., Major and O’Brien, 2005) have described a range of social identity adjustments that are crucial for the well-being of stigmatized groups members (Bobowik et al., 2014). Outgroup derogation (e.g., prejudice) is one strategy. Individual upward mobility from a discriminated group to another more privileged one is another (see Tausch et al., 2015). Furthermore, recent studies on negative identity management have proposed that disadvantaged ethnic minority members cope by “navigating” *multiple* group identities (e.g., Curtin et al., 2016). Multiple identities are described in those studies as resources, not replacing, but rather repositioning and reinterpreting the disadvantaged (ethnic) identity in light of other group memberships. In particular, members of ethnic minorities can experience a psychological overlap between their exclusive (i.e., ethnic) and inclusive (i.e., national) identities. Some studies have found that this particular configuration of their “collective” identity drives support for minority activism (e.g., Çakal et al., 2016; Curtin et al., 2016). Note that such identity reconfigurations communicate societal changes minority members wish to see (Smith et al., 2015), for example a normative change in ethnic (or racial) groups differentiation. In the present study, we consider the interplay of ethnic and national identifications when examining the relationship between contact and activism.

### National Identity and Its Mobilizing Effect for Ethnic Minorities

National identification has been conceptualized as a relational construct, which “provides a means of appealing to a group of people [...] within a given territory” (Reicher and Hopkins, 2001, p. 26). As national subgroups, ethnic minorities can thus identify with, that is feel attached to, both ethnic and national groups. Furthermore, “native” or traditional ethnic minorities may strongly identify with the nation considered their ancestral homeland (Sibley and Liu, 2007). Indeed, the strength of ethnic minorities’ national identification has been shown to vary across countries (e.g., Staerklé et al., 2010).

As ethnic minorities can simultaneously identify with an ethnic and a national group, we argue that when interpreting the sedating effect of positive intergroup contact on minority activism via reduced ethnic identification national identification should be accounted for. Recent research supports such reframing. For example, national identification can increase minority members’ expectations to be treated fairly and help them believe in social change (Chryssochoou and Lyons, 2011). As a result, when unequal treatment is experienced, national identification fuels minority members’ disappointment (Klandermans et al., 2008). National identification also fosters feelings of entitlement to political rights among immigrant minority members (Klandermans et al., 2008; Scuzzarello, 2015) as well as claims of country “ownership” among established minorities (Brylka et al., 2015). Additionally, minorities can strategically navigate identities (e.g., adapt their statements of identification) as a function of the audience they are communicating with. Ethnic minority members may make more national identification claims before a host country audience compared to an audience of ethnic peers (Barreto et al., 2003). Furthermore, a particular overlapping of ethnic and national self-definitions labeled *dual identity* (i.e., identifying as both a member of the ethnic minority and of the national group) has been shown to uniquely predict support for ethnic minority activism (e.g., Simon and Ruhs, 2008; Simon and Grabow, 2010; see also Verkuyten, 2016, for the effects of normative contexts in this process). For example, in the US, Glasford and Dovidio (2011) found that ethnic minority members exposed to a dual identity representation (as both American and member of an ethnic group) were more motivated to address disparities during intergroup interactions compared to participants exposed to a common ingroup representation (as solely American). The mobilizing effect of ethno-national identification stresses an important feature of minority activism: when minority group members become active, they do so because their minority identity is defined in terms integrating the “more inclusive societal context in which this struggle has to be fought out” (Simon and Klandermans, 2001, p. 319; see also van Zomeren, 2016).

To summarize, national identification is a dynamic identity process observed among minority members, alongside ethnic identification. Our reading of research on intergroup contact, negative social identity management strategies, and collective identity explanations of group activism (see also Çakal et al., 2011) suggests that the synergy of ethnic identification—involving separation and grievances—and national identification—bringing entitlement—more accurately reflects the collective identification of ethnic minority members and should thus be considered when examining the sedating effects of positive contact. The goal of the current study is thus to examine whether the negative effect of positive intergroup contact on ethnic activism via reduced ethnic identification is buffered by the national identification of minority members.

**FIGURE 1:**
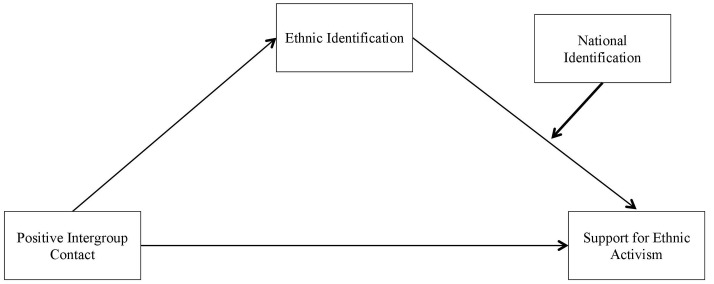
**The hypothesized moderated mediation model predicting ethnic activism**.

We make a novel theoretical contribution by examining national identification as a buffering factor of the demobilization process resulting from intergroup contact. Reasoning in terms of identity (re)configurations allows considering identification as a dynamic process rather than a fixed ethnic identity (see Tajfel and Turner, 1979). As prior research has shown the importance of multiple identities for endorsement of social change, in particular the combination of ethnic and national identities, we suggest that national identification counteracts the sedative effects of positive contact. Based on the outlined theoretical arguments, in our research in Bulgaria, we examined the moderating role of national identification in the relationships between contact, ethnic identification and ethnic activism. In particular, we hypothesize a moderated mediation pattern underlying the sedative effect of intergroup contact (see **Figure 1**): the mediation pattern from intergroup contact to reduced Roma activism through Roma’s weakened ethnic identification should be moderated by identification with the Bulgarian nation. Identification as a member of the Bulgarian nation should thus buffer the sedative effect of experiencing positive contact with members of the Bulgarian national majority. We expect that the indirect effect from intergroup contact to activism through ethnic identification emerges only for Roma with low national identification, or at least is stronger for them than for Roma with high national identification.

### The Context of the Present Study: Roma in Bulgaria

We also make novel empirical contributions to intergroup contact and collective action literature by examining the perspective of the Roma minority in Bulgaria. Roma are an understudied group, despite being historically and currently the most severely rejected ethnic minority in Europe (Heath and Richards, 2016). The study of negative identity management among Roma is thus particularly important. However, others perceive Roma as an ethnicity more than Roma themselves (Csepeli and Simon, 2004). In other words, the Roma ethnicity is formed by discrimination experiences and attributed stereotypes as least as much as by a particular appearance, language or ancestry (Kligman, 2001). Moreover, Roma are spread all over Europe forming small national minorities that self-identify with the various national groups or traditions (Marushiakova and Popov, 2007). Accordingly, Roma minorities are an interesting case for the study of collective identity predictors of activism, because both the abstract and externally defined ethnic identity and the various nation-specific minority identities can be potentially mobilized.

In Bulgaria, the Roma minority has experienced assimilation policies before, during and after the communist regime (Marushiakova and Popov, 2008a). Such policies have certainly also affected the self-identification of Roma across generations. Indeed research has shown that Bulgarian Roma and ethnic Bulgarian adolescents do not differ in their level of attachment to the nation (Dimitrova et al., 2014). After the political transition to democracy, Bulgaria declared itself a multicultural country and highlighted the different cultural and religious groups in its constitution. Compared to neighboring countries also with large Roma minorities (e.g., Hungaria, Romania), contemporary Bulgaria is the only country with a national strategy for integration of Roma people in its legislation (European Commission against Racism and Intolerance, 2014). Yet, the upsurge of ethnic nationalism in Eastern Europe along with economic difficulties throughout the European Union have reached Bulgaria and constitute a renewed threat to Roma inclusion. Roma communities are scapegoated (see, e.g., Petkov, 2006), subjected to ethnic characterization and systematic othering (Kostova et al., 2011), and relegated to a sub-proletarian class (Vassilev, 2004). In this context, support for Roma activism against persisting ethnic discrimination is a highly relevant, albeit sensitive, enterprise. These observations pointing to an integrative normative context coupled with ethnic stigmatization call for a nuanced perspective when studying the relationship between contact with the Bulgarian majority and Roma activism (see also Kauff et al., in press for cross-national evidence). Roma’s identity management and adjustments related to ethnic and national identity need to be considered (see Reysen et al., 2016 for a similar approach of the Roma issue).

## Materials and Methods

### Participants

Three-hundred-twenty self-declared Roma from Bulgaria participated in this study.^1^ A two-stage sampling procedure was used, which consisted of first determining sampling points in both urban and rural areas, and then seeking eight respondents stratified by age and gender from each sampling point. Respondents were recruited by a Bulgarian survey agency in two districts of the country: Montana (north-west) and Stara Zagora (center). The final sample consisted of 162 females and 158 males respondents (*M*_age_ = 43.30 years, *SD* = 16.66, range = 15–83 years old) nested in 40 clusters (i.e., sampling points). All respondents reported Bulgarian as their first language with the exception of one participant who reported Romani. Regarding educational level, 15.3% of Bulgarian Roma had never been to school or not completed primary education. The large majority (60.3%) of respondents had primary or lower secondary education. The proportion of respondents having an upper secondary education was 23.1%, whereas 1.2% had a university degree. Note that those with upper secondary and university education were slightly overrepresented in our sample as compared to official figures (Pamporov and Kabakchieva, 2012). We also asked participants to define what was their own or their family’s current economic situation on a scale ranging from 1 (*We have enough money for our needs and are able to save*) to 5 (*We have to cut back on consumption and we don’t manage on our earnings*). The subjective economic situation of Roma in our sample was modest (*M* = 4.03, *SD* = 0.97), with only 7.8% or respondents reporting having enough for their needs.

### Procedure and Measures

The data used in this study are part of a research project examining social psychological processes underlying interethnic attitudes and prejudice of both the Bulgarian majority and of the two largest ethnic minorities (Roma and Turks) in contemporary Bulgaria. The survey questionnaires were designed in English and then translated into Bulgarian using a back-translation method. They were administered face to face by professional interviewers, who were members of the national majority. This survey was carried out in accordance with the recommendations of the Code of Deontology of the Swiss Psychological Society and of the American Psychological Association, and in compliance with the Law for Protection of Personal Data in Bulgaria. Respondents were provided with the necessary information for informed consent, as well as guaranteed anonymity and right to withdraw from the survey at any time.^2^

**Table 1** summarizes means, standard deviations, and correlations for the final variables used in the subsequent analyses.

**Table 1:** Means and correlations for close positive contact, ethnic and national identification and ethnic activism (*N* = 320 Roma respondents).

	*M*	*SD*	1	2	3
(1) Close positive contact^a^	0.00	0.90	–	–	–
(2) Ethnic identification	4.26	0.79	-0.19^∗∗∗^	–	–
(3) National identification	3.82	1.03	0.08	0.17^∗∗^	–
(4) Ethnic activism	4.13	0.88	0.16^∗∗^	0.19^∗∗∗^	0.29^∗∗∗^

*^a^Standardized score; ^∗∗^p < 0.01; ^∗∗∗^p ≤ 0.001.*

We assessed *close positive contact* with three items (see Voci and Hewstone, 2003; Pettigrew et al., 2007) addressing the quantity and quality of Bulgarian Roma’s close contact with ethnic Bulgarians (*“How many ethnic Bulgarians do you know well?”* and *“How often do you experience these encounters with ethnic Bulgarians you know well as pleasant?”*) and extended contact *(“How many people you know have ethnic Bulgarian friends?”*). Quantity of contact (direct and extended) was rated on a scale ranging from 1 (*None*) to 4 (*Many*), whereas quality of contact was rated on a scale ranging from 1 (*Never*) to 5 (*Always*). The respondents reported both a lot of close contacts with ethnic Bulgarians (*M* = 3.38, *SD* = 0.95) as well as knowing many Bulgarian Roma having ethnic Bulgarian friends (*M* = 3.40, *SD* = 0.85). Since the quality item was filtered for the respondents reporting no contact with ethnic Bulgarians, we considered these respondents “never” having positive contact and replaced the missing values (*n* = 34) accordingly (i.e., with 1). Respondents perceived their encounters with ethnic Bulgarian as generally positive (*M* = 3.65, *SD* = 1.24). Extended contact was strongly correlated with quantity (*r* = 0.73, *p* < 0.001) and quality of contact (*r* = 0.64, *p* < 0.001). Moreover, a principal component analysis yielded a single factor explaining 80% of variance. Thus, the three items were considered as representing a single concept, that is, close positive contact with ethnic Bulgarians. Due to different response scales, the three items were standardized prior to computing a close positive contact score (α = 0.88; see also Pettigrew et al., 2007 for an intergroup friendship index using both direct and extended contacts).

*Ethnic and national identification* were measured with three parallel items (*“Do you often think of yourself as* a member of the Bulgarian nation/Roma?” “*Is being* part of the Bulgarian nation/Roma *important to you?” “Do you feel close to other* members of the Bulgarian nation/Roma?,” e.g., Sidanius et al., 1997). The scale ranged from 1 (*No, not at all*) to 5 (*Yes, very much*). Both identification scales reached good or adequate reliability (for national identification α = 0.84, for ethnic identification α = 0.69). Both high level of ethnic and national identification were reported in the sample though ethnic identification was significantly higher than national identification, *t*(319) = −6.77, *p* < 0.001. The two identification indices were moderately correlated.

We assessed *ethnic activism* with four items (α = 0.85; see collective action scale of Mähönen and Jasinskaja-Lahti, 2015) addressing the willingness to improve the position of Roma in Bulgaria by different political contributions (*vote for a candidate defending Roma’s rights; defending the rights of Roma in public debate; defending the rights of Roma in situations where you notice discrimination; taking part in cultural events organized by Roma*).^3^ The five-point scale ranged from 1 (*No, not at all)* to 5 (*Yes, very much)*.

## Results

The analytic strategy was to first attempt to replicate the sedating effect of intergroup contact on ethnic activism through reduced ethnic identification, and then to test whether national identification can buffer this effect. Preliminary analyses revealed that, due to the clustered nature of the sampling (i.e., with eight respondents from the same sampling point), data is non-independent (*ICC* of ethnic activism = 0.50). We therefore tested path models using the Mplus Complex command, which allows accounting for the non-independence and non-normality of observations (Muthén and Muthén, 1998–2010, p. 533).^4^ Ethnic and national identification indices were centered as their interaction was modeled in the second step of the analysis. Gender, age, educational level, and perceived economic situation of the respondents were controlled for.

First, we assessed the mediating role of ethnic identification in the relationship between close positive contact and ethnic activism. As expected, we replicate the sedating effect of contact. The left panel (A) of **Table 2** shows that contact was negatively associated with ethnic identification, which in turn was positively associated with ethnic activism. The indirect effect of contact on ethnic activism through ethnic identification was significant (*B* = −0.04, *SE* = 0.02, *p* = 0.044).^5^ However, also an unexpected positive relationship between positive close contact and ethnic activism was observed. The only significant effect of the control variables was a positive relationship between educational level and ethnic activism (*B* = 0.11, *SE* = 0.05, *p* = 0.044).

**Table 2:** Unstandardized coefficients and standard errors of the mediation model and of the moderated mediation model.

	(A) Mediation analysis				(B) Moderated mediation analysis			
	Ethnic identification (Me)		Ethnic activism (Y)		Ethnic identification (Me)		Ethnic activism (Y)	
	*B*	*SE*	*B*	*SE*	*B*	*SE*	*B*	*SE*
Close positive contact (X)	−0.16^∗^	0.06	0.17^∗^	0.08	−0.16^∗^	0.06	0.14^†^	0.07
Ethnic identification (Me) centered			0.27^∗∗^	0.09			0.20^∗∗^	0.08
National identification (Mo) centered							0.19^∗∗^	0.08
Ethnic × National (Me × Mo)							−0.16^∗^	0.08
*R*^2^	0.05^∗^	0.03	0.11^∗^	0.05	0.05^∗^	0.03	0.17^∗∗^	0.07

*X, independent variable; Me, mediator variable; Mo, moderating variable; Y, dependent variable. Gender, age, educational level and perceived economic situation of the respondents were controlled for in the analyses.*

*^†^p = 0.054; ^∗^p < 0.05; ^∗∗^p ≤ 0.01.*

**FIGURE 2:**
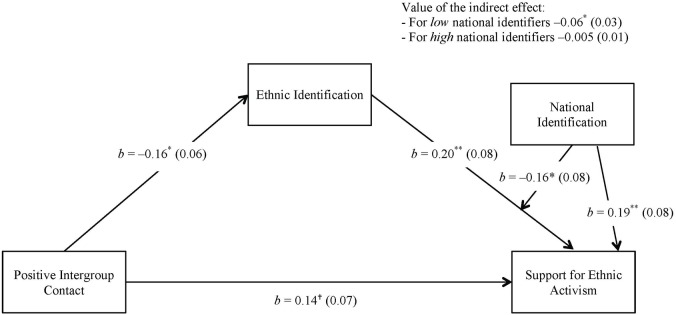
**The empirical path model predicting ethnic activism.** ^†^*p* = 0.054; ^∗^*p* < 0.05; ^∗∗^*p* = 0.01.

Next, we tested our moderated mediation hypothesis. Close positive contact was entered as the independent variable (X), ethnic identification as mediator (Me) and ethnic activism as the dependent variable (Y). The direct path from close positive contact to ethnic activism was also estimated. National identification was specified as the variable moderating (Mo) the relationship between ethnic identification and ethnic activism. The findings are summarized in the right panel (B) of **Table 2** and depicted in **Figure 2**. Again the sedating effect of contact was revealed: close positive contact was related to reduced ethnic identification, which in turn was positively associated with ethnic activism. National identification was positively related to ethnic activism. Importantly, national identification moderated the relationship between ethnic identification and activism. As predicted, the indirect effect of close contact on ethnic activism mediated by reduced ethnic identification was buffered by national identification. The sedating effect was significant for low national identifiers (*B* = −0.06, *SE* = 0.03, *p* = 0.042), while not for high national identifiers (*B* = −0.005, *SE* = 0.01, *p* = 0.678). Positive contact sedated activism thus only for Bulgarian Roma who reported weak self-identification as a member of the Bulgarian nation.

The direct path from close contact to ethnic activism remained marginally significant and positive in the moderated mediation model. None of the control variables yielded significant effects. Finally, we also examined whether national identification moderates the path between close positive contact with the majority and ethnic identification (i.e., the first stage of the mediation), or the direct path from close positive contact to ethnic activism. Additional moderated mediation analyses revealed that neither one of these paths was moderated by national identification. This suggests the specificity of interplaying ethnic and national identification that predict support for minority activism.

## Discussion

The aim of the present study was to disentangle the sedating effect of positive contact with the majority on ethnic minority activism, by considering not only the reduction of ethnic identification, but the synergy of ethnic and national identification. Our chief contribution was to show that national identification buffered this sedating process: the demobilization effect of contact through reduced ethnic identification was found only among individuals with low levels of national identification.

The revealed buffering effect of national identification clearly confirms the need to consider more than one identity when studying minority activism (see also Curtin et al., 2016). This finding also suggests the existence of a non-conforming style of national identification among ethnic minority members. As Moscovici (1976) proposed, minorities use productive – as opposed to sedative – forms of conformity and alignment with the social context. This is in line with research on the effects of dual identity on ethnic activism (e.g., Simon and Grabow, 2010), which shows that the motor of ethnic minorities’ struggle is a collectively endorsed commitment to the higher societal level, which in turn results in an entitlement for claims in the name of the subordinate ethnic group. Furthermore, we chose to focus on national identification among other potentially relevant interplaying identities (e.g., religious, opinion-based, gender) for several reasons. The demonstration that Roma identify with the nation contributes to contesting the stereotype of a “nomadic” and stateless minority fundamentally different from other ethnic minorities. Indeed, the Roma are often seen as “forever pilgrims,” defined as a non-territorial and transnational ethnic community (Vermeersch, 2003). Roma should be rather compared to Indigenous people with regard to attachment to the national homeland: though their ethnic group identity derives from family lineages rooted in a homeland, national majorities consider them as unassimilated in the modern nation state. Furthermore, national identification can provide this kind of internally segmented minorities the psychological and political resources to challenge intergroup disparities (see Çakal et al., 2016). In contrast to Indigenous people, Roma have personal experiences of state paternalism under the communist rule, or at least they have been exposed to nostalgic discourses about the less marked ethnic segregation back then as compared to the present. These considerations suggest that national identification is an important component of Roma collective identity.

Our results also revealed an unexpected positive direct relationship between positive close contact and ethnic activism, which was not moderated by national identification. Several studies have indeed highlighted that positive interethnic contact does not exempt experiencing negative emotions due to threat of persistent interethnic prejudice (e.g., Shelton et al., 2005). Contact with the advantaged group provides opportunities for personally assessing intergroup injustice and leading minority members to realize their disadvantaged position. For example, Poore et al. (2002) revealed that Inuits’ perception of group-based discrimination increased as a function of their contacts with the North-American culture. This result does not contradict our main finding of national identification buffering the sedative effect of contact, but simply suggests other parallel consequences of intergroup encounters.

### The Interplay of Ethnic and National Identification in the Bulgarian Context

The findings of this research require reflecting on the Bulgarian Roma’s national identification that implies a common ingroup shared with ethnic Bulgarians. The demobilizing effect of contact has been originally studied in segregated societies (e.g., South Africa, USA, Israel). In those contexts, bringing people back together involves the development of a common ingroup identity that can overlap with national identity, and the abandon of ethnic (or racial) categories. In contrast, post-socialist Bulgaria has experienced an inverse normative shift, from implicit interethnic differences to more explicit interethnic recognitions. During the communist regime in Bulgaria, interethnic contact took place within an ethno-nationalist ideology affirming equality of citizens whatever their ethnic origin, and state policies were implemented ensuring the public integration of Roma citizens (Marushiakova and Popov, 2008b). After the transition to democracy and to market economy, the Bulgarian nationalist discourse and constitution highlighted multiculturalism. Ethnic minority members were encouraged to develop a strong identification as a member of the nation “colored” with ethnicity, as Pettigrew (2010) commented about the specificity of interethnic relations in Bulgaria. Today, the inherited ethno-nationalist model is reinterpreted through political and media discourse describing Bulgarian poverty and economic difficulties as having a specific “racial” origin: Roma (see Kligman, 2001). One must note though that Bulgaria is praised for having avoided any major interethnic clash.

Accordingly, Bulgarian Roma’s feeling of national belonging may not reflect a by-product of their positive relationships with the majority, but rather suggest a reminiscence of, or even a battle for, access to a national group from which they are materially and symbolically excluded (Pereira and Green, 2017). This interpretation echoes research in the field of coping with social disadvantage. According to system justification theory (Jost et al., 2004), members of socially disadvantaged groups engage in a struggle for social change only when the unfavorable nature of their self- or group-image “overcomes the strength of system justification needs and tendencies” (p. 887). This claim supports our interpretation, since the image of the Roma as a national subgroup is unquestionably unfavorable. The negotiation of both ethnic identification—stigmatized but nevertheless protected by the Bulgarian constitution—and identification as a marginalized member of the nation results in an unfavorable collective identity. Likewise, Barreto and Ellemers (2009) proposed that identifying with an under-represented or culturally threatened group accentuates rather than sedates the perception of social disadvantage. Finally, Hopkins and Blackwood (2011) demonstrated that stigmatized minority members (i.e., Muslims in Britain), who have internalized the denial of their citizenship, sometimes actively claim citizenship as a form of resistance against others’ negative assumptions about their identities. Future research should substantiate these interpretations of the combative nature of national identification among Roma people in Bulgaria and in other East European countries with somewhat different legislative frameworks regarding ethnic minorities.

Moreover, the sedating effect of positive contact with the majority revealed in this study (among low national identifiers) may be due to the legacy of implicit interethnic boundaries combined with exclusion from the current legitimate national group (i.e., ethnic Bulgarians). In a study conducted in England among marginal citizens of Roma origin, Casey (2014) found a tendency to favor the status quo, that he argued was a strategy for maintaining Roma ethnic identity and cultural traditions that have survived *thanks to* social exclusion. Additionally, it is likely that contemporary Bulgarian Roma experience some degree of political cynicism (due to corruption, prolonged discrimination, etc.), known to impede activism (Klandermans et al., 2008). Further studies should unravel whether political cynicism is relevant in Roma minorities’ struggle for social equality. More generally, to better understand what predicts ethnic minority activism, we must consider how intergroup contact between minorities and majorities (along with the normative context in which it takes place) constructs the interethnic issue at stake in the first place. Eastern European countries provide a fascinating field to study how minorities (and majorities) use their ethnic and national belonging to adapt to recent social change, and eventually readjust and react to the new emerging inequalities.

Some limitations of our study must be acknowledged. First, the wording of the national identification measure (i.e., being member of the Bulgarian nation) might have triggered the interplay of sub- and superordinate identities. This wording was, however, the best alternative in this context: asking Roma respondents to what extent they felt “Bulgarian” could come across as either insulting (i.e., as Bulgarian Roma have no other homeland, they may perceive that the interviewer sees them as authorized or unauthorized immigrants) or misleading (i.e., some Roma subgroups claim to be “ethnic” Bulgarians because of their lineage with proto-Bulgarian people living on the national territory before the modern state constitution). Second, one should not underestimate the bias in self-reporting intergroup contact and support for ethnic activism, in particular as the interviewers were members of the national majority. Confronted to a non-Roma audience, survey respondents may have made national identification claims (see Barreto et al., 2003). Third, the support for ethnic activism in our sample does not reflect the actual implementation of Roma activism in Bulgaria. Other predictors of engagement in activism should be accounted for in future research on Roma activism. In addition to support for collective activism, more individual responses to social disadvantage or the combination of individual and collective strategies should be considered (see Becker et al., 2015). Finally, no firm causal conclusions regarding the relationships between variables can be drawn from our cross-sectional design. Still, a large number of experimental studies have established that collective identity and intergroup contact are important predictors of activism increasing our confidence in the current findings. In our view, studying activism among a harshly stigmatized, yet understudied group such as Roma offsets the caveat of being unable to make firm causal claims.

## Conclusion

This study contributes to the scarce body of knowledge about Roma’s activism (see, e.g., Vermeersch, 2014; Reysen et al., 2016 for other recent studies). We revealed that Roma activism in Bulgaria was shaped by contact with the advantaged national majority as well as by an interplay of ethnic and national identification forming a collective Bulgarian Roma identity that was mobilized to support social change. We demonstrated that the previously evidenced sedating effect of contact on activism due to a reduced ethnic identification can be counteracted by identifying with the Bulgarian nation. Our results and interpretations stress the adaptive (and instrumental) role of identification to both a subordinate ethnic and a superordinate national group, which need to be considered within the specific societal structures (van Zomeren, 2016). Rather than being fixed realities, ethno-national identities are collective identities, construed through communication and interactions, that vary across contemporary nations.

## Author Contributions

AP, EG, and EV developed the study concept and designed the research. AP and EV performed the data analysis and interpretation under the supervision of EG. AP and EG drafted the manuscript, and EV provided critical revisions.

## Conflict of Interest Statement

The authors declare that the research was conducted in the absence of any commercial or financial relationships that could be construed as a potential conflict of interest.

## Footnotes

1 Our target sample size (determined by the fieldwork budget as well as statistical considerations) was 300 Bulgarian Roma respondents. We anticipated model designs with 10–15 parameters and bore in mind suggestions for sample sizes of 20 times the number of parameters (e.g., Kline, 2015). The final sampling point grid allowed for a sample of 320 Bulgarian Roma respondents in the entire survey involving national majority and minority members.

2 The questionnaire for the Roma sample included other measures that were not considered in the current study. The order of questionnaire items was the following: ethnic and national identification, ideological orientations, intergroup contact with and prejudice toward ethnic Bulgarians, intergroup contact with and prejudice toward Bulgarian Turks, social distance, status perceptions, perceived discrimination, acculturation orientations and expectations, political behavior and ethnic activism, and socio-demographics.

3 The item on the willingness to participate in cultural events organized by Roma could be seen as measuring a social creativity strategy (i.e., coping) rather than as an active resistance strategy (Becker, 2012). However, the item was strongly correlated with the other ethnic activism items (*r*s > 0.44, *p*s < 0.001). Furthermore, a principal component analysis confirmed that the four items loaded onto a single factor explaining 69% of variance.

4 We also conducted the same analyses using the PROCESS macro (Hayes, 2013) and tested indirect effects with bootstrapping. The result patterns were identical. However, PROCESS does not take into account the nested structure of data, necessary for the current sample.

5 The indirect effects, estimated with Mplus, are calculated as the product between the regression coefficient of the IV on the mediator and the regression coefficient of the mediator on the DV.
